# Pollinator-Mediated Isolation May Be an Underestimated Factor in Promoting Homoploid Hybrid Speciation

**DOI:** 10.3389/fpls.2016.01183

**Published:** 2016-08-04

**Authors:** Yongpeng Ma, Renchao Zhou, Richard Milne

**Affiliations:** ^1^Key Laboratory for Plant Diversity and Biogeography of East Asia, Kunming Institute of Botany, Chinese Academy of SciencesKunming, China; ^2^State Key Laboratory of Biocontrol and Guangdong Provincial Key Laboratory of Plant Resources, Sun Yat-sen UniversityGuangzhou, China; ^3^Institute of Molecular Plant Sciences, School of Biological Sciences, University of EdinburghEdinburgh, UK

**Keywords:** entomophilous plant, flower color, homoploid hybrid speciation, pollinator mediated isolation, reproductive isolation

It is widely acknowledged that allopolyploidy via hybridization and genome doubling can easily lead to speciation, as polyploid hybrids can be immediately isolated from diploid parental taxa due to high levels of sterility caused by uneven numbers of chromosome complements in their progeny (Soltis and Soltis, 2009; Abbott et al., 2013). However, homoploid hybrid speciation (HHS), in which a hybrid lineage becomes genetically isolated from their parents and functions as a distinct evolutionary unit, has been thought to be fairly uncommon (Chase et al., 2010; Servedio et al., 2013). Schumer et al. (2014) proposed three criteria for proving HHS: evidence of hybridization in the genome, reproductive isolation (RI) of hybrid lineages from parental species, and evidence that this reproductive isolation is a consequence of hybridization. According to these criteria, only one case of HHS in sunflowers was strictly documented in plants till now (Schumer et al., 2014). These criteria highlight two reasons why HHS is harder to detect and prove than allopolyploid speciation: the first is the lack of chromosome number change, whereas the second is that molecular evidence for hybridization becomes harder to detect as the parent species become more closely related, and it is known that HHS tends to involve more closely related parent species than allopolyploidy events (Abbott et al., 2013).

Inconsistent with the low rate of documented HHS events in plants, a recent simulation study published in *PLOS Genetics* by Schumer et al. (2015) predicts that homoploid hybrids could rapidly become isolated from parental species by fixing combinations of genes that hinder successful reproduction with parental species, even in the presence of substantial ongoing immigration from parental species to the new homoploid hybrid species. The main reason for the discrepancy might lie in the fact that most population genetics studies are merely focused on evidence of hybridization in the genome, without examining the strength of RI which can be assessed by other integrative methods. Pollination biology approach can address the extent of RI between hybrids and parental species. For example, the detailed examination of flowering time difference and the pattern of pollen flow mediated by pollinator behavior quantified by pollen DNA barcoding method could provide the strength of pre-pollination RI between parental species and hybrids whereas post-pollination RI could be assessed by comparing pollen tube growth and reproductive fitness between parental species and hybrids (e.g., fruit set, seed set, seed viability) using artificial pollination (e.g., Ma et al., 2014; Bell et al., 2016; Ma et al., 2016). After the confirmation of certain traits that originate from hybridization and are responsible for RI, QTL can be then applied to link the variation of traits to specific genetic loci. It should be pointed out that abiotic ecological factors could promote RI by long-term geographic isolation between hybrids and parents. In this commentary, however, we focus on flowering plants with an entomoplily system that could sometimes build up RI between hybrids and parents by biotic factors with a relative short time.

It is a common phenomenon in entomophilous plants that post-zygotic RI like hybrid inviability and/or sterility is not strong enough to select against all the hybrid progeny. Early-generation hybrids with morphological intermediacy are likely to be less effective in attracting the pollinator(s) of either parent, given long term interaction between and adaptive evolution of flower characters of parental species and pollinators (Chase et al., 2010). Thus, strong selection against hybrids with intermediate morphologies would be expected to occur frequently. In addition, even where certain pollinators are shared between hybrids and parental species, theory predicts that once F1s are casually formed, backcrosses to either or both parental species should frequently occur, therefore hybrids can be easily re-absorbed by parental species (Arnold, 1997). In rare cases where F1s dominate hybrid zones, selection against second generation hybrids appears to prevent both interspecific gene flow and the possible creation of homoploid hybrid species (Milne et al., 2003; Milne and Abbott, 2008).

However, entomoplily in some flowering plants seems much more complicated than theoretical predictions suggest, because of highly specific interactions between plants and pollinators. Interestingly, three recent studies on pollination biology have indicated that HHS in entomophilous plants should not be so uncommon. The first case involves pollinator mediated RI between the homoploid hybrid species *Penstemon clevelandii* and its parental species, by flower color shift due to hybridization (Wolfe et al., 1998). The magenta flower color of *P. clevelandii* has established RI by selection for a distinct bee and hummingbird pollination syndrome, whereas one of its parental species has red flower and is pollinated by hummingbirds, and the other has lavender flower and is pollinated by wasps (Wolfe et al., 1998). The second well documented case is *Iris nelsonii*, a diploid species that originated from hybridization between *I. fulva, I. hexagona*, and *I. brevicaulis.* It is isolated from one of its progenitors (*I. hexagona*) by attracting hummingbirds due to large red flowers, while *I. hexagona* is mainly pollinated by bumblebees due to blue flowers, although other isolation barriers such as habitat and environmental isolation are also present (Taylor et al., 2013). The two study cases mentioned above show a partial shift of pollinator preferences creating pre-zygotic RI in the pollination process, due to flower color differentiation as a result of hybridization. The latest case involves *Narcissus* hybrids which recruit novel pollinators under natural conditions (Marques et al., 2016). Their results showed that the hybrids not only emitted new floral volatiles, but also recruited new pollinators of ants, which were never witnessed in their parental species. This provides strong evidence for pollinator mediated RI between hybrids and parental species (Marques et al., 2016).

Overall, after molecular confirmation of hybridization, work remains to be done for examining RI between hybrids and their parents and further testing if the characters responsible for RI originate from hybridization. Recent studies on entomophlilous plants, albeit only three cases, have proved the power of homoploid hybridization in creating new traits responsible for RI. We therefore emphasize the need for field investigation of pollinator mediated RI after hybridization is confirmed by molecular data for entomophlilous plants. We believe that HHS may not be uncommon in flowering plants, especially in many genera in which hybridization is thought to play an important role in their evolution and speciation; the rarity of proven examples likely reflects difficulty of detection. Integrative approaches to investigating potential cases should examine the possibility of pollinator-mediated isolation from parental species, and how this might have been generated during hybridization. The Sino-Himalaya region, where many congeneric species occur sympatrically and are interfertile, is an ideal place for conducting such studies.

## Author contributions

YM conceived and wrote the paper, RZ and RM revised the paper. All authors approved it for publication.

## Funding

This study was supported by the National Science Foundation of China-Yunnan (Grant no. 1302262) and Fundamental Research Funds for the Central Universities (15lgjc23).

### Conflict of interest statement

The authors declare that the research was conducted in the absence of any commercial or financial relationships that could be construed as a potential conflict of interest.
